# HLA Class I (A and B) Allele Polymorphism in a Moroccan Population Infected with Hepatitis C Virus

**DOI:** 10.3390/cimb46120842

**Published:** 2024-12-13

**Authors:** Safa Machraoui, Abdelmalek Hakmaoui, Khaoula Errafii, Mehdi Knidiri, Lamiaa Essaadouni, Khadija Krati, Brahim Admou

**Affiliations:** 1 Laboratory of Immunology and Human Leukocyte Antigen, Center of Clinical Research, Mohammed VI University Hospital, Marrakech 40080, Morocco; hakmaoui15@yahoo.fr (A.H.); br.admou@uca.ac.ma (B.A.); 2 Biosciences Research Laboratory, Faculty of Medicine and Pharmacy, Cadi Ayyad University, Marrakech 40080, Morocco; la.essaadouni@uca.ac.ma; 3 African Genome Center, Mohammed VI Polytechnic University (UM6P), Ben Guerir 43151, Morocco; khaoula.errafii@um6p.ma (K.E.); m.knidiri70@gmail.com (M.K.); 4 Gastroenterology Department, Arrazi Hospital, Mohammed VI University Hospital Center, Marrakech 40000, Morocco; kratielidrissi@gmail.com

**Keywords:** hepatitis C virus, HLA-A, HLA-B, polymorphism, Morocco

## Abstract

Hepatitis C virus (HCV) infection is one of the major health burdens worldwide. Its course depends on the virus itself and the host’s immune responses. The latter are conditioned by immunogenetic factors, in particular human leukocyte antigens (HLAs), whose role in determining the outcome of infection varies according to populations and ethnic groups. The current study attempted to investigate the possible relationship between HLA-A and HLA-B allele polymorphism and its impacts on the clinical outcome of HCV for a better understanding of disease susceptibility and clearance. A cross-sectional and comparative study was carried out on 40 patients with hepatitis C and 100 ethnically matched healthy control subjects originating from southern Morocco. HLA class I alleles were typed using the high-resolution PCR-SSO method. The prevalence of certain HLA class I alleles differed significantly between HCV-infected individuals and healthy controls. In particular, HLA-A*02:01 was less prevalent in chronic HCV infection (*p* = 0.002), indicating a potential protective effect, while the higher prevalence of HLA-A*68:02, A*66:01 B*15:03, B*41:02, B*44:03, and B*50:01 in patients could indicate a predisposing factor. These findings support the association of these immunogenetic markers with HCV infection, indicating their possible role in determining clinical and genotype forms as well as the outcome of HCV infection. Thus, an in-depth analysis of these alleles could lead to a better understanding of HCV pathogenesis and potential targeted interventions.

## 1. Introduction

Hepatitis C virus (HCV) infection is a global health problem. The World Health Organization (WHO) estimates that 50 million people are living with chronic HCV, which is the leading cause of liver cancer and is responsible for approximately 242,000 deaths each year. Early detection and treatment are therefore essential to prevent serious complications [1]. One of the most alarming aspects of hepatitis C is its long-term impact on human health. Many people infected with the virus remain unaware of their condition for years, as the disease often has no noticeable symptoms in its early stages. This silent progression can lead to chronic hepatitis C, which can eventually lead to liver cirrhosis, liver failure, or hepatocellular carcinoma (HCC) [2,3,4].

The role of human leukocyte antigen (HLA) in anti-infectious immune responses is indisputable, particularly during HCV infection, making this complex system an important immunogenetic determinant of its evolution [5,6].

Indeed, the ability of the immune system to recognize and react to viral infections largely depends on HLA molecules [7], which, by exposing viral antigens to cytotoxic T lymphocytes (CTLs), modulates the host immune response, enabling the clearance or persistence of the virus [8]. Furthermore, HLA-A and -B allele diversity can potentially be used as a predictor for HCV susceptibility and progression [9], while this variability may contribute to the control of HCV infection. In fact, certain HLA alleles have been associated with more effective immune responses against HCV, notably, HLA-B*27, -B*57, -B*8, -A*02, and -A*03, leading to viral clearance [10,11,12,13,14]. In contrast, several studies have demonstrated the association between HLA variety and HCV outcomes, highlighting the involvement of certain HLA alleles in HCV infection and progression, notably HLA-A*9 and -A*6 [15,16].

Polymorphisms in HLA class I are very crucial for regulating the characteristics of immune responses to chronic liver diseases and, depending on the type of liver injury, might influence the progression of liver fibrosis or the development of HCC [17]. Some specific HLA alleles, such as HLA-A*02:01, have protective effects on viral persistence and fibrogenesis, whereas others, such as HLA-B*08:01, are associated with increased chronic inflammation and fibrosis susceptibility [18]. Within the context of immunological clearance against HCC, these polymorphisms may influence antigen presentation, allowing tumor cells with some risk-increasing alleles to evade the immune response [17]. Understanding these relationships is critical for identifying at-risk patients and customizing immunotherapeutic strategies.

According to the literature, the frequency and the HLA specificities of such linkage may vary depending on the ethnic background of the population [10,19,20], which can identify genetic markers of treatment responsiveness and disease prognosis, enabling improved medical interventions in a diverse genetic landscape.

The main objective of this study was to investigate the relationship between HCV infection and HLA-A and -B allele polymorphism in a Moroccan population compared to healthy individuals.

## 2. Materials and Methods

### 2.1. Study Design

A cross-sectional and comparative study was carried out on 40 HCV-infected patients and 100 healthy controls enrolled during a period of two years (2022–2024) in collaboration with the gastroenterology department of the University Hospital. Patients and controls are from the southern regions of Morocco.

Control individuals are potential organ or stem cell donors; they are clinically healthy, with negative HCV, HBV, and HIV serologies. Additionally, controls had no history of chronic liver disease or known exposure to HCV or other bloodborne pathogens.

Patients with HCV infection were included based on predefined inclusion criteria. All patients were confirmed as positive for HCV by molecular biology. The cohort was then divided into two distinct groups based on treatment response and disease severity. The first group included 26 patients with chronic HCV infection, confirmed by a high viral load, assayed at 2 intervals (3 and 6 months after treatment), and a fibroscan score between F3 and F4. The second group consisted of 14 patients that were deemed to have attained successful clearance of the virus, as evidenced by 3- and 6-month post-treatment assessments of significantly decreased viral loads, indicative of a sustained virologic response, and minimal liver damage fibroscan scores between F0 and F1. Patients who did not meet the diagnostic criteria for HCV infection and those co-infected with HBV or HIV, as well as those who did not consent to participate in the study, were excluded.

### 2.2. Ethical Considerations

The study was approved by the Medical Ethics Committee of Mohammed VI University Hospital in Marrakech, and informed consent was obtained from each patient prior to enrolment in the study. The controls investigated in this study correspond to those obtained as a part of the routine activity of the HLA laboratory. Sociodemographic data were extracted anonymously from medical records for patients and from the HLA laboratory computer database for controls, under the supervision of the head of the gastroenterology department and the HLA laboratory manager, respectively. In this case, informed consent was not necessary.

### 2.3. Sample Collection and Processing

Peripheral venous blood was collected in two 5 mL tubes containing Ethylenediaminetetraacetic Acid (EDTA) as an anticoagulant in accordance with the protocol designed for HLA typing. This bifurcated approach ensures the stabilization and preservation of different cell types pertinent for comprehensive HLA typing. Upon collection, samples were immediately and carefully transferred to the laboratory. Transportation was conducted under rigorous conditions using specialized sample transport bags designed to maintain sample integrity during transit. This immediate transfer is critical to prevent degradation and to ensure the preservation of the cellular components necessary for accurate HLA typing.

DNA extraction: Genomic DNA extraction from peripheral blood mononuclear cells (PBMCs) was executed using the QIAmp DNA Mini kit (Qiagen, Hilden, Germany), following a multi-step protocol. Briefly, this process began with cell lysis, where PBMCs were treated with a lysis buffer containing chaotropic salts to disrupt cell membranes and release genomic DNA. The lysate was then applied to a silica-based column, where the genomic DNA was selectively bound to the silica membrane, allowing impurities to be washed away. A series of ethanol-based wash buffers were used to cleanse DNA, ensuring the removal of salts, metabolites, and other contaminants. Finally, the purified DNA was eluted from the membrane using an elution buffer, carefully releasing the DNA without causing damage. The resulting DNA was of high quality, as confirmed by quantitative and qualitative assessments using a NanoDrop^TM^ 2000/2000c Spectrophotometer (Thermo Scientific^TM^, Waltham, MA, USA), which measured DNA concentration and purity, critical for downstream molecular analyses.

### 2.4. HLA Typing Methodology

HLA class I typing was conducted using Reverse SSO DNA typing (Thermo Fisher Scientific, Labtype^TM^ XR, Waltham, MA, USA), a high-resolution method based on Luminex xMAP technology. The DNA samples underwent PCR amplification, with the aim of amplifying particular portions of HLA genes associated with class I (A, B). PCR products were amplified and then hybridized to the probe-coated beads in an assay-optimized environment. The Luminex system was used to detect the microspheres’ fluorescent signals. The color coding of each microsphere corresponds to a specific HLA allele probe, and the intensity of the fluorescence indicates the hybridization strength, allowing us to identify the HLA alleles present in the sample.

Data analysis was conducted using Fusion software version 4.1.0, which integrates fluorescence intensity data from the Luminex xMAP technology. This software utilizes a library of known allele sequences to match the probe fluorescence patterns observed, allowing for high-resolution determination of HLA typing.

This high-resolution typing is critical for assessing HLA allele distribution among the HCV-infected patients and healthy controls, providing insights into genetic susceptibility and resistance to HCV infection.

### 2.5. Statistical Analysis

Chi2 and Fisher’s exact tests were employed to compare allele frequencies inside the two groups. Probability values were determined using two-sided testing and were considered statistically significant when *p* ≤ 0.05. In order to analyze the HLA disease association, we filtered out alleles that strongly deviated from HWE (alleles with an HWE *p*-value below 0.05) and the logistic regression approach was employed using MiDAS package in R version 1.11.1 [21]. The HLA evolutionary divergence (HED) between alleles of a single individual was computed for each genotype at HLA-A and -B loci using the HLAdivR package in R version R-4.4.2 [22].

## 3. Results

### 3.1. Descriptive Analysis of Laboratory Characteristics of Moroccan Patients with HCV Infection

Among the 40 patients included in this study, 55% of them were female (sex ratio = 0.909). The mean age was 55.4 years ± 13. Demographic and relevant clinical and laboratory characteristics of the patients are presented in Table 1.

Source of infection: As shown in Table 1, the most common reported source of HCV infection was blood transfusion, accounting for 42.5% of cases, followed by dental care, with 25% of cases, while for 30% of cases, the cause of transmission remains untraced.

HCV genotype profiles: The distribution of HCV genotypes in patients revealed a predominance of genotype 1b, noticed in 47.5% of cases, followed by genotype 2, with 27.5% of cases, and then 2a, which accounted for 12.5%, while genotype 1a was found in only 2.5% of the cases (Table 1).

Fibroscan scores: The severity of liver damage and fibrosis in patients as assessed by fibroscan scores showed that most of them (32.5%) had mild fibrosis, corresponding to F0–F1 stages, followed by moderate fibrosis (F2–F3) observed in 27.5% of cases, while 17.5% had severe fibrosis (F3–F4). A small group (5%) had fibrosis scores ranging from F1 to F2. Furthermore, 17.5% of the patients exhibited unclear fibrosis phases, implying poor diagnostic assessments in some situations.

### 3.2. Distribution of HLA Class I Genes Among Patients with HCV Infection and Controls

Among the main HLA class I allele groups found in our populations, A2 displayed a significantly lower frequency in HCV-infected patients (10%) than in healthy controls (32.5%) (*p* = 0.0002), while A23 and A68 were more frequent in the HCV-infected group, with 11.25% for both versus 4% (*p* = 0.0001) and 4.5% (*p* = 0.0002) in controls, respectively (Table 2).

Conversely, the A30 and B50 allele groups were more frequent in HCV-infected patients (12.5% and 17.5%, respectively) than in healthy controls (7.5% and 7.5%, respectively), with *p*-values of 0.0008 and <0.0001, respectively. Similarly, despite their low prevalence, other allele groups were significantly more frequent in HCV-infected patients than in controls, like A1 (*p* = 0.0111), A24 (*p* = 0.0097), and B53 (*p* = 0.0077) (Table 2).

### 3.3. Distribution of HLA-A and -B Alleles in HCV-Infected Patients and Controls

By comparing HLA-A and HLA-B allele frequencies between patients and controls, we noted that the HLA-A*02:01 allele was significantly less frequent in HCV patients, at 7.50% vs. 32.5% in healthy controls (*p* = 0.002338), suggesting a protective effect of this allele from HCV infection, while the HLA-A*68:02 allele was found in 5% of patients and was absent in the control group (*p* = 0.013). The same applies to HLA-B*35:01, observed in 1.25% of patients compared to 7.5% in controls (*p* = 0.022).

In contrast, HLA-A*66:01 was found in 6.25% of patients versus 2.5% of controls, with a significant *p*-value of 0.0191, thus indicating a potential predisposition of HLA-A*66:01 to chronic HCV infection.

No significant difference was observed for the frequency of HLA-A*01:01, HLA-A*03:01, and HLA-A*24:02 between the two groups, with 11.25%, 5%, and 8.75% in patients vs. 9.5% (*p* = 0.666), 5% (*p* = 0.431), and 5.5% (*p* = 0.325) in controls, respectively (Table 3).

HLA-B*07:02 was present in 7% of controls and absent in patients with a *p*-value of 0.0169. Similarly, HLA-B*15:03 was detected in 5% of patients and 1.5% of controls (*p*-value = 0.0394), and both HLA-B*44:03 and HLA-B*50:02 exhibited high frequencies in the patient group, estimated at 3.75% (*p* = 0.039), hence implying a potential predisposition of these alleles to chronic HCV infection.

On the other hand, there was a non-significant difference between patients and controls regarding the frequency of HLA-B*14:01, HLA-B*27:01, and HLA-B*49:01 alleles, for which the *p*-values were 0.1678, 0.533, and 0.343, respectively (Table 4).

Each locus was tested against the Hardy–Weinberg (H–W) equilibrium. The *p*-values at both loci are above the typical threshold for significance of *p* > 0.05 (*p* = 0.808 and 0.861 for HLA-A and -B loci, respectively) (Table 5), and therefore, there is no significant deviation from the Hardy–Weinberg equilibrium. This indicates that both HLA-DRB1 and HLA-DQB1 allele frequencies in the sample population are balanced, which could probably be a sign of a stable population without selective pressure on these loci in association with HCV infection. This result supports the assumption that the alleles observed in the present sample reflect the genetic structure of the larger population with respect to these loci.

### 3.4. HLA Class I Allele Frequencies of HCV-Infected Subjects

This study compares HLA class 1 allele frequencies in people who cleared the hepatitis C virus and those who developed chronic hepatitis C. Among the various alleles studied, B*50:01 has a strong correlation (*p* = 0.004) with chronic HCV, with levels significantly higher in chronic patients (21.15%) than in the clearance group (1%). While alleles like A*01:01 and A*23:01 had higher frequencies in the viral clearance group, they did not achieve statistical significance.

### 3.5. Evolutionary Divergence of HLA-A and -B in Patients and Controls

**Figure 1:**
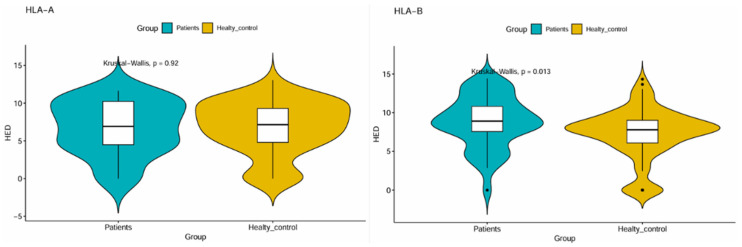
Human leucocyte antigen evolutionary divergence of HLA-A and -B in patients and controls.

The assessment of HLA evolutionary divergence (HED) for the HLA-A and HLA-B allele groups in patients and healthy controls, as illustrated by the violin plots overlaid with inner boxplots (Figure 1), shows that the distribution of HED scores is similar between patients and controls for HLA-A.

The distribution of HED scores is wide in both groups, suggesting variability even within each group. However, the median HED score seems to be virtually the same between HCV-infected patients and healthy controls, at 6.911 and 7.140, respectively.

For the HLA-A allele groups, the Kruskal–Wallis test gave evidence of a lack of a significant difference in HED scores between patients versus controls (*p* = 0.92).

For the HLA-B allele groups, strong differential distributions were observed between patients and controls, as illustrated by Figure 1, revealing a tighter cluster that shifted slightly to the left in the patient group compared to their healthy counterparts. Also, a higher median HED score was noticed in patients, but not in healthy controls. The Kruskal–Wallis test yielded a *p*-value of 0.013 (Figure 1), demonstrating a significant difference in the distributions of HED scores between the two groups.

## 4. Discussion

Our study represents one step on the path to better understanding the potential impact of HLA-A and B alleles on HCV infection regarding the genetic diversity among different ethnic populations. This should be considered when determining the infection outcome and therapeutic strategies.

Our investigations highlighted notable differences in the frequency of multiple HLA genes, with statistical significance for A23, A30, A68, B50, and B51 between a group of Moroccan HCV-infected patients and matched healthy controls. In contrast, the A2 and B7 allele groups were present at lower frequencies in the HCV-infected group. It is worth noting that some of these HLA genes such as HLA-A23, A68, and B50 are relatively less common in Moroccan individuals, at less than 5%.

Using high-resolution four-digit HLA genotyping, providing more accurate and robust data, the current study revealed differences in HLA allele frequency between patients with HCV infection and healthy controls, suggesting an immunogenetic background that may influence the outcome of HCV infection. Interestingly, among HLA class I alleles present at a higher frequency in healthy controls, HLA-A*02:01 achieved significance after correction (*p* = 0.00004, adjusted *p* = 0.0023) and a positive estimate (0.138), which might suggest a potential increase in protection from disease when this allele is present. Actually, this allele is hypothesized to be more efficient at presenting viral epitopes than other alleles, resulting in a higher cytotoxic response of virus-specific CD8+ T cells [23]. However, HLA-A*02 manifests opposing trends in different ethnic groups: a risk for Caucasians and protection for non-Caucasians [24]. Another study highlights the potential of HLA-A*02:01 as an enhanced innate ability to spontaneously clear HCV in a cohort of Chinese voluntary blood donors [23].

Thanks to the high-resolution four-digit genotyping method used in our study, allowing us to obtain more accurate and robust data than the standard two-digit level used in earlier studies [17,18,19], the results show that only A*02:01 was significantly associated with HCV clearance. Such a specific association would not have been highlighted by using only a two-digit HLA genotyping approach.

On the other hand, the higher prevalence of HLA-A*68:02 and A*66:01 in patients may indicate a possible predisposing factor, but they did not reach significance after Bonferroni correction. Thus, investigation of a larger and more diversified population is needed to validate and extend these preliminary results.

Regarding the differences in HLA-B alleles between patients and controls and their potential functional relevance in the immune response against HCV, HLA-B*07:02 was found to be more common in controls than in patients without reaching significance after correction. In contrast, HLA-B*15:03, B*41:02, B*44:03, and B*50:02 might exhibit a predisposing tendency. In addition, several investigations have shown that HLA-B*57 is a predictor of HCV spontaneous clearance [12,25,26]. This association was noticed in our study when comparing healthy controls to HCV-infected patients (*p* = 0.0204), as shown in Table 2. Furthermore, our important findings might reveal candidates for further investigation, possibly through replication in larger cohorts or through studies involving anti-HCV(+)/HCV RNA(−) cohorts within the general population, with the aim of confirming their effects on persistence or protection against HCV infection. However, HLA-B*27 has been strongly associated with spontaneous viral clearance in multiple studies [14,27].

The high incidence of the B*50:01 allele in the chronic HCV group (Table 6) implies an explanation for disease persistence in the Moroccan population, emphasizing the allele’s potential role in reducing the host’s ability to clear the virus. This observation is consistent with current evidence that implicates distinct HLA alleles in the modulation of immunological responses to HCV, changing the trajectory of acute to chronic infection [28]. An Egyptian study showed evidence of viral clearance by expressing the B*50 allele [29]. Alleles like A*01:01 and A*23:01 had higher frequencies in the viral clearance group, but they did not achieve statistical significance.

It has been hypothesized that heterozygous HLA individuals have a greater variety of peptides to present to T lymphocytes than homozygous individuals. As a result, they are more able to elicit a specific immunological response [30,31,32]. Moreover, the advantage of HLA allelic diversity can enhance the functional capacity for peptide antigen presentation, thereby increasing protection against pathogens through the ability to activate protective T-cell immunity [33].

The HLA-A locus does not vary as a risk factor for the disease according to the results of patients having significant differences in HED scores compared with healthy donors. This can imply that the role of the HLA-A locus is not critical in relevant conditions or not fully represented by HLA Evolutionary Divergence. In contrast, the fact that the HED scores for the HLA-B locus were significantly different across patients and controls suggests that this locus may have an impact on immune response or disease susceptibility. The higher median HED score in patients may represent the selection for greater genetic variation at this locus in response to disease management, or it could reflect genetic predispositions that influence disease development or severity.

Variations in HED scores among distinct populations or within patient groups may elucidate evolutionary pressures or associations with disease, as reported by Sanchez-Mazas A [34], who noted that particular HLA alleles correlating with elevated HED scores might provide either protective effects or predisposition to specific medical conditions. In clinical practice, the definition of HED profiles at HLA-A and HLA-B loci can guide the formulation of disease management and prevention strategies, including the development of vaccines, for which immunogenetic diversity and hence immune responses could significantly improve efficacy in heterogeneous populations [34,35].

## 5. Conclusions

The current study investigated the relationship between HLA class I allele polymorphisms and the clinical course of HCV infection in Moroccan patients. HLA-A*02:01 was strongly present across a high-frequency distribution in healthy controls when compared to HCV-infected patients. The high incidence of the B*50:01 allele in the chronic HCV group when compared to the viral clearance group emphasizes the allele’s potential role in reducing the host’s ability to clear the virus.

The identification of specific HLA alleles linked to HCV infection outcome has substantial implications for public health policies in countries like Morocco. The implementation of HLA-based genetic screening could identify populations at risk for developing chronic HCV infection, thereby facilitating timely interventions, with appropriate strategies for both treatment and prevention, which in turn may improve clinical outcomes and minimize healthcare costs.

However, it is necessary to validate and extend these preliminary results to larger and more diversified populations, using longitudinal studies, combining assessment of T-cell immune responses and clinical evolution, and considering all parameters that may interfere with the course of the disease.

## Figures and Tables

**Table 1:** Clinical and biological characteristics of patients with hepatitis C virus infection.

Characteristics	Values
Male/Female (%)	45/55
Age (mean ± SD)	55.4 ± 13
**Source of infection (%)**	
Sharp equipment	2.5
Blood transfusion	42.5
Dental care	25
Unknown source	30
**HCV genotype (%)**	
1b	47.5
1a	2.5
2	27.5
2a	12.5
Not defined	1
**Fibroscan score (%)**	
F0-F1	27.5
F1-F2	5
F2-F3	32.5
F3-F4	17.5
Not defined	17.5

**Table 2:** Distribution of HLA specificities between HCV-infected patients and controls.

HLA Allele Groups	HCV-Infected Patients (N = 40)	Controls (N = 100)	HCV-Infected Patients vs. Controls
	Frequency (%)	Frequency (%)	*p*-Value
**A1**	11.25	9.5	**0.0111**
**A2**	10	32.5	**0.0002**
A3	8.75	8.5	0.0587
A11	1.25	3	-
**A23**	11.25	4	**0.0001**
**A24**	8.75	5.5	**0.0097**
A26	3.75	6	0.7105
A29	2.5	4	0.6730
**A30**	12.5	7.5	**0.0008**
A31	3.75	1	0.0669
A32	0	5	0.3213
**A33**	5	2	**0.0204**
A34	0	2.5	-
**A66**	6.25	2	**0.0063**
**A68**	11.25	4.5	**0.0002**
A69	0.5	0	-
A74	2.5	1	0.1900
A80	1.25	1	0.4839
B7	2.5	7	-
B8	3.75	8	-
B13	0	0	-
B14	3.75	3	0.3494
**B15**	7.45	2	**0.002**
B18	3.75	4	0.4005
B27	1.25	2	-
B35	2.5	7.5	-
B37	2.5	0	0.0773
**B38**	6.25	5	0.0734
B39	1.25	2.5	-
**B40**	2.5	5	-
**B41**	5	1	**0.0074**
B42	0	2	-
**B44**	10	8	**0.0149**
B45	1.25	8	0.4447
B47	0	1	-
B48	0	0	-
**B49**	5	5.5	0.1510
**B50**	17.5	7.5	**<0.0001**
**B51**	8.75	11.5	0.1900
B52	1.25	0	0.2806
**B53**	5	1.5	**0.0077**
B55	0	0.5	-
**B57**	5	2	**0.0204**
B58	3.75	2	0.1344
B73	0	0.5	-
B78	0	1	-

Allele groups with statistical significance after Fisher’s exact correction are indicated in bold.

**Table 3:** HLA-A allele frequencies in patients with HCV infection and matched healthy controls.

HLA-A Alleles	HCV-Infected Patients %	Controls %	*p*-Value	*p*-Adjusted	Confidence Interval
01:01	11.25	9.5	0.66699	-	-
**02:01**	7.50	32.5	**0.00004**	0.002338 *	(0.04885 to 0.338)
02:02	1.25	-	0.34335	-	-
02:05	1.25	-	0.34335	-	-
03:01	5	8.5	0.43104	-	-
03:02	1.25	-	0.34335	-	-
03:05	1.25	-	0.34335	-	-
03:60	1.25	-	0.34335	-	-
11:01	1.25	3	0.66699	-	-
23:01	10	4	0.27367	-	-
23:30	1.25	-	0.34335	-	-
24:02	8.75	5.5	0.32561	-	-
26:01	3.75	6	0.55053	-	-
29:01	-	4	0.16787	-	-
29:02	1.25	-	0.34335	-	-
29:72	1.25	-	0.34335	-	-
30:01	5.00	4	-	-	-
30:02	6.25	3.5	0.28080	-	-
30:04	1.25	-	0.34335	-	-
31:01	3.75	1	0.34219	-	-
32.01	-	5	0.09657	-	-
33:01	5	2	0.23589	-	-
34:01	-	2.5	0.30167	-	-
66:01	6.25	2	**0.01910**	**NS**	(0.861 to 14.55)
68:01	6.25	4.5	0.55172	-	-
68:02	5	-	**0.01322**	**NS**	(0.752 to 13.44)
69:01	-	0.5	0.60873	-	-
74:01	2.5	1	0.60873	-	-
80:01	1.25	1	0.34335	-	-

* Alleles with statistical significance after Fisher’s exact correction are indicated in bold. NS: not significant

**Table 4:** Distribution of HLA-B alleles in patients with HCV infection and healthy controls.

HLA-B Alleles	HCV-Infected Patients %	Controls %	*p*-Value	*p*-Adjusted	Confidence Interval
**07:02**	-	7	**0.0169**	**NS**	**-**
07:05	2.5	-	0.1169	-	-
08:01	3.75	8	0.2745	-	-
14:01	-	3	0.1678	-	-
**14:02**	3.75	-	**0.0394**	**NS**	**-**
15:01	-	0.5	-	-	-
**15:03**	5	1.5	**0.0394**	**NS**	(0.757 to 18.99)
15:17	1.25	-	0.3433	-	-
15:31	1.2	-	0.3433	-	-
18:01	3.75	4	0.6939	-	-
27:01	-	2	0.5530	-	-
27:05	1.25	-	0.3433	-	-
**35:01**	1.25	7.5	**0.0222**	**NS**	(0.008 to 0.826)
35:02	1.25	-	0.3433	-	-
37:01	2.5	-	0.1169	-	-
38:01	6.25	5	0.5517	-	-
39:01	-	2.5	0.3016	-	-
39:06	1.25	-	0.3433	-	-
40:01	1.25	5	0.1701	-	-
40:02	1.25	-	0.3433	-	-
41:01	-	1	-	-	-
41:02	5	1	**0.0132**	**NS**	**-**
42:01	-	2	0.3016	-	-
44:02	5	7.5	0.5870	-	-
44:03	3.75	0.5	**0.0394**	**NS**	**-**
44:09	1.25	-	0.3433	-	-
45:01	1.25	8	0.0558	**NS**	(0.00732 to 0.696)
47:01	-	1	0.5471	-	-
49:01	3.75	5.5	-	-	-
49:70	1.25	-	0.3433	-	-
50:01	13.75	7.5	0.3050	NS	(0.872 to 5.198)
50:02	3.75	-	**0.0399**	**NS**	**-**
51:01	6.25	11.5	0.1759	-	-
51:09	2.5	-	0.1169	-	-
52:01	1.25	-	0.3433	-	-
53:01	5	1.5	0.2358	-	-
55:01	-	0.5	-	-	-
57:01	3.75	1	0.3421	-	-
57:02	1.25	1	-	-	-
58:01	3.75	2	0.4157	-	-
73:01	-	0.5	-	-	-
78:01	-	1	-	-	-

Alleles with statistical significance after Fisher’s exact correction are indicated in bold. NS: not significant.

**Table 5:** Hardy–Weinberg equilibrium test results.

Locus	Observed	Expected	Chi-Square	*p*-Value
HLA-A	36	37.49	0.06	0.808
HLA-B	39	37.92	0.03	0.861

**Table 6:** HLA class I allele frequencies of HCV-infected subjects.

HLA Class 1 Alleles	Viral Clearance	Chronic Hepatitis C	*p*-Value
	Frequency (%)	Frequency (%)	
A*01:01	17.86	7.69	0.234
A*02:01	14.29	3.85	0.159
A*02:02	-	1.92	1
A*02:05	3.57	-	0.35
A*03:01	3.57	5.77	1
A*03:02	-	1.92	1
A*03:05	-	1.92	1
A*03:60	-	1.92	1
A*11:01	-	1.92	1
A*23:01	14.29	7.69	0.322
A*23:30	3.57	-	0.35
A*24:02	10.71	7.69	0.679
A*26:01	5.77	5.77	0.539
A*29:02	1.92	1.92	1
A*29:72	3.57	-	0.35
A*30:01	3.57	5.77	1
A*30:02	3.57	7.69	1
A*30:04	-	1.92	1
A*31:01	-	5.77	0.539
A*33:01	7.14	3.85	0.602
A*66:01	3.57	7.69	0.64
A*68:01	7.14	5.77	1
A*68:02	3.57	5.77	1
A*74:01	-	3.85	0.533
A*80:01	-	1.92	1
B*07:05	-	3.85	0.533
B*08:01	7.14	1.92	0.276
B*14:02	-	5.77	0.539
B*15:03	3.57	5.77	1
B*15:17	3.57	-	0.35
B*15:31	3.57	-	0.35
B*18:01	-	5.77	0.533
B*27:05	-	1.92	1
B*35:01	-	1.92	1
B*35:02	3.57	-	0.35
B*37:01	3.57	1.92	1
B*38:01	3.57	7.69	0.64
B*39:06	3.57	-	0.35
B*40:01	3.57	-	0.35
B*40:02	3.57	1.92	1
B*41:02	3.57	5.77	1
B*44:02	10.71	1.92	0.115
B*44:03	3.57	3.85	1
B*44:09	3.57	-	0.35
B*45:01	-	1.92	1
B*49:01	7.14	1.92	0.276
B*49:70	3.57	-	0.35
B*50:01	1	21.15	0.004 *
B*50:02	7.14	1.92	0.276
B*51:01	3.57	7.69	0.64
B*51:09	7.14	-	0.117
B*52:01	-	1.92	1
B*53:01	-	7.69	0.278
B*57:01	7.14	1.92	0.276
B*57:02	-	1.92	1
B*58:01	7.14	1.92	0.276

* Alleles with statistical significance after Fisher’s exact correction are indicated in bold.

## Data Availability

The data used in this study are not publicly available due to privacy restrictions. Access to the data is restricted in accordance with the ethical guidelines and regulations governing the protection of participant confidentiality and privacy. However, researchers interested in replicating or verifying the findings presented in this study may request access to the data through the appropriate institutional review board. Requests for data access will be considered on a case-by-case basis, subject to approval by the relevant authorities and compliance with applicable privacy regulations. For inquiries regarding data access, please contact Pr. Brahim ADMOU/Clinical research Center/Mohammed VI University Hospital Center at br.admou@uca.ac.ma.

## References

[1] WHO (2023). Hepatitis C [Internet]. https://www.who.int/news-room/fact-sheets/detail/hepatitis-c.

[2] Alter M.J. (2007). Epidemiology of hepatitis C virus infection. World J. Gastroenterol..

[3] Ghany M.G., Strader D.B., Thomas D.L., Seeff L.B. (2009). Diagnosis, management, and treatment of hepatitis C: An update. Hepatology.

[4] World Health Organization (2016). Guidelines for the Screening, Care and Treatment of Persons with Chronic Hepatitis C Infection [Internet].

[5] Spaan M., Janssen H.L.A., Boonstra A. (2012). Immunology of hepatitis C virus infections. Best Pract. Res. Clin. Gastroenterol..

[6] Rhodes S.L., Erlich H., Im K.A., Wang J., Li J., Bugawan T., Jeffers L., Tong X., Su X., Rosen H.R. (2008). Associations between the human MHC and sustained virologic response in the treatment of chronic hepatitis C virus infection. Genes Immun..

[7] Bowen D.G., Walker C.M. (2005). Adaptive immune responses in acute and chronic hepatitis C virus infection. Nature.

[8] Smith S., Honegger J.R., Walker C. (2021). T-Cell Immunity against the Hepatitis C Virus: A Persistent Research Priority in an Era of Highly Effective Therapy. Cold Spring Harb. Perspect. Med..

[9] Crux N.B., Elahi S. (2017). Human Leukocyte Antigen (HLA) and Immune Regulation: How Do Classical and Non-Classical HLA Alleles Modulate Immune Response to Human Immunodeficiency Virus and Hepatitis C Virus Infections?. Front. Immunol..

[10] Huang J., Xu R., Wang M., Liao Q., Huang K., Shan Z., You Q., Li C., Rong X., Fu Y. (2019). Association of HLA-DQB1*03:01 and DRB1*11:01 with spontaneous clearance of hepatitis C virus in Chinese Li ethnicity, an ethnic group genetically distinct from Chinese Han ethnicity and infected with unique HCV subtype. J. Med. Virol..

[11] Cangussu L.O.F., Teixeira R., Campos E.F., Rampim G.F., Mingoti S.A., Martins-Filho O.A., Gerbase-DeLima M. (2011). HLA class II alleles and chronic hepatitis C virus infection. Scand. J. Immunol..

[12] Chuang W.C.M., Sarkodie F., Brown C.J., Owusu-Ofori S., Brown J., Li C., Navarrete C., Klenerman P., Allain J.P. (2007). Protective effect of HLA-B57 on HCV genotype 2 infection in a West African population. J. Med. Virol..

[13] Salloum S., Oniangue-Ndza C., Neumann-Haefelin C., Hudson L., Giugliano S., aus dem Siepen M., Nattermann J., Spengler U., Lauer G.M., Wiese M. (2008). Escape from HLA-B*08-Restricted CD8 T Cells by Hepatitis C Virus Is Associated with Fitness Costs. J. Virol..

[14] Neumann-Haefelin C., Timm J., Schmidt J., Kersting N., Fitzmaurice K., Oniangue-Ndza C., Kemper M.N., Humphreys I., McKiernan S., Kelleher D. (2010). Protective effect of human leukocyte antigen B27 in hepatitis C virus infection requires the presence of a genotype-specific immunodominant CD8+ T-cell epitope. Hepatology.

[15] Matzaraki V., Kumar V., Wijmenga C., Zhernakova A. (2017). The MHC locus and genetic susceptibility to autoimmune and infectious diseases. Genome Biol..

[16] Kumar V., Kato N., Urabe Y., Takahashi A., Muroyama R., Hosono N., Otsuka M., Tateishi R., Omata M., Nakagawa H. (2011). Genome-wide association study identifies a susceptibility locus for HCV-induced hepatocellular carcinoma. Nat. Genet..

[17] Hofmann M., Tauber C., Hensel N., Thimme R. (2021). CD8+ T Cell Responses during HCV Infection and HCC. J. Clin. Med..

[18] Pei R., Zhang W., Wang S., Huang X., Zou Y., Wang G. (2022). Prognostic Value of HLA Class I in Patients with Hepatocellular Carcinoma. Clin. Lab..

[19] El-Bendary M., Neamatallah M., Elalfy H., Besheer T., Kamel E., Mousa H., Eladl A.H., El-Setouhy M., El-Gilany A.H., El-Waseef A. (2019). HLA Class II-DRB1 Alleles with Hepatitis C Virus Infection Outcome in Egypt: A Multicentre Family-based Study. Ann. Hepatol..

[20] Arrieta-Bolaños E., Hernández-Zaragoza D.I., Barquera R. (2023). An HLA map of the world: A comparison of HLA frequencies in 200 worldwide populations reveals diverse patterns for class I and class II. Front. Genet..

[21] midasHLA.pdf [Internet]. https://new.bioconductor.org/packages/release/bioc/manuals/midasHLA/man/midasHLA.pdf.

[22] rbentham/HLAdivR: Calculate Diversity Scores Between HLA Class I Alleles Version 1.0.0 from GitHub [Internet]. https://rdrr.io/github/rbentham/HLAdivR/.

[23] Huang J., Huang K., Xu R., Wang M., Liao Q., Xiong H., Li C., Tang X., Shan Z., Zhang M. (2016). The Associations of HLA-A∗02:01 and DRB1∗11:01 with Hepatitis C Virus Spontaneous Clearance Are Independent of IL28B in the Chinese Population. Sci. Rep..

[24] Wang J.H., Zheng X., Ke X., Dorak M.T., Shen J., Boodram B., O’Gorman M., Beaman K., Cotler S.J., Hershow R. (2009). Ethnic and geographical differences in HLA associations with the outcome of hepatitis C virus infection. Virol. J..

[25] Kim A.Y., Kuntzen T., Timm J., Nolan B.E., Baca M.A., Reyor L.L., Berical A.C., Feller A.J., Johnson K.L., Schulze zur Wiesch J. (2011). Spontaneous control of HCV is associated with the expression of HLA-B*57 and preservation of targeted epitopes. Gastroenterology.

[26] Asher A.K., Santos G.M., Evans J., Dokubo E.K., Lee T.H., Martin J.N., Deeks S.G., Tobler L.H., Busch M., Hunt P.W. (2013). Human leukocyte antigen B*57 does not fully explain hepatitis C clearance in HIV controllers. AIDS.

[27] McKiernan S.M., Hagan R., Curry M., McDonald G.S.A., Kelly A., Nolan N., Walsh A., Hegarty J., Lawlor E., Kelleher D. (2004). Distinct MHC class I and II alleles are associated with hepatitis C viral clearance, originating from a single source. Hepatology.

[28] Lapa D., Garbuglia A.R., Capobianchi M.R., Del Porto P. (2019). Hepatitis C Virus Genetic Variability, Human Immune Response, and Genome Polymorphisms: Which Is the Interplay?. Cells.

[29] Zekri A.R.N., El-Mahallawy H.A., Hassan A., El-Din N.H.A., Kamel A.M. (2005). HLA alleles in Egyptian HCV genotype-4 carriers. Egypt. J. Immunol..

[30] Lima B.A. (2024). Calculated Human Leucocyte Antigens Evolutionary Divergence (cHED). OBM Transplant..

[31] Frater A.J., Brown H., Oxenius A., Günthard H.F., Hirschel B., Robinson N., Leslie A.J., Payne R., Crawford H., Prendergast A. (2007). Effective T-Cell Responses Select Human Immunodeficiency Virus Mutants and Slow Disease Progression. J. Virol..

[32] Archbold J.K., Macdonald W.A., Gras S., Ely L.K., Miles J.J., Bell M.J., Brennan R.M., Beddoe T., Wilce M.C., Clements C.S. (2009). Natural micropolymorphism in human leukocyte antigens provides a basis for genetic control of antigen recognition. J. Exp. Med..

[33] Arora J., Pierini F., McLaren P.J., Carrington M., Fellay J., Lenz T.L. (2020). HLA Heterozygote Advantage against HIV-1 Is Driven by Quantitative and Qualitative Differences in HLA Allele-Specific Peptide Presentation. Mol. Biol. Evol..

[34] Sanchez-Mazas A. (2020). HLA studies in the context of coronavirus outbreaks. Swiss Med. Wkly..

[35] Piertney S.B., Oliver M.K. (2006). The evolutionary ecology of the major histocompatibility complex. Heredity.

